# Economic Burden of Community-Acquired Antibiotic-Resistant Urinary Tract Infections: Systematic Review and Meta-Analysis

**DOI:** 10.2196/53828

**Published:** 2024-10-09

**Authors:** Nina Jiayue Zhu, Misghina Weldegiorgis, Emma Carter, Colin Brown, Alison Holmes, Paul Aylin

**Affiliations:** 1 National Institute for Healthcare Research, Health Protection Research Unit in Healthcare-Associated Infection and Antimicrobial Resistance, Imperial College London, London, United Kingdom; 2 Centre for Antimicrobial Optimisation, Imperial College London, London, United Kingdom; 3 Healthcare Associated Infections, Fungal, Antimicrobial Resistance, Antimicrobial Use, and Sepsis Division, UK Health Security Agency, London, United Kingdom; 4 Institute of Systems, Molecular and Integrative Biology, University of Liverpool, Liverpool, United Kingdom; 5 Department of Primary Care and Public Health, School of Public Health, Imperial College London, London, United Kingdom; 6 Patient Safety Translational Research Centre, Institute of Global Health Innovation, Imperial College London, , London, United Kingdom, United Kingdom

**Keywords:** cost-effectiveness, urinary tract infection, antibiotic resistance, mortality, hospital length of stay

## Abstract

**Background:**

Antibiotic resistance (ABR) poses a major burden to global health and economic systems. ABR in community-acquired urinary tract infections (CA-UTIs) has become increasingly prevalent. Accurate estimates of ABR’s clinical and economic burden are needed to support medical resource prioritization and cost-effectiveness evaluations of urinary tract infection (UTI) interventions.

**Objective:**

This study aims to systematically synthesize the evidence on the economic costs associated with ABR in CA-UTIs, using published studies comparing the costs of antibiotic-susceptible and antibiotic-resistant cases.

**Methods:**

We searched the PubMed, Ovid MEDLINE and Embase, Cochrane Review Library, and Scopus databases. Studies published in English from January 1, 2008, to January 31, 2023, reporting the economic costs of ABR in CA-UTI of any microbe were included. Independent screening of titles/abstracts and full texts was performed based on prespecified criteria. A quality assessment was performed using the Integrated Quality Criteria for Review of Multiple Study Designs (ICROMS) tool. Data in UTI diagnosis criteria, patient characteristics, perspectives, resource costs, and patient and health economic outcomes, including mortality, hospital length of stay (LOS), and costs, were extracted and analyzed. Monetary costs were converted into 2023 US dollars.

**Results:**

This review included 15 studies with a total of 57,251 CA-UTI cases. All studies were from high- or upper-middle-income countries. A total of 14 (93%) studies took a health system perspective, 13 (87%) focused on hospitalized patients, and 14 (93%) reported UTI pathogens. *Escherichia coli*, *Klebsiella pneumoniae*, and *Pseudomonas aeruginosa* are the most prevalent organisms. A total of 12 (80%) studies reported mortality, of which, 7 reported increased mortality in the ABR group. Random effects meta-analyses estimated an odds ratio of 1.50 (95% CI 1.29-1.74) in the ABR CA-UTI cases. All 13 hospital-based studies reported LOS, of which, 11 reported significantly higher LOS in the ABR group. The meta-analysis of the reported median LOS estimated a pooled excess LOS ranging from 1.50 days (95% CI 0.71-4.00) to 2.00 days (95% CI 0.85-3.15). The meta-analysis of the reported mean LOS estimated a pooled excess LOS of 2.45 days (95% CI 0.51‐4.39). A total of 8 (53%) studies reported costs in monetary terms—none discounted the costs. All 8 studies reported higher medical costs spent treating patients with ABR CA-UTI in hospitals. The highest excess cost was observed in UTIs caused by carbapenem-resistant Enterobacterales. No meta-analysis was performed for monetary costs due to heterogeneity.

**Conclusions:**

ABR was attributed to increased mortality, hospital LOS, and economic costs among patients with CA-UTI. The findings of this review highlighted the scarcity of research in this area, particularly in patient morbidity and chronic sequelae and costs incurred in community health care. Future research calls for a cost-of-illness analysis of infections, standardizing therapy-pathogen combination comparators, medical resources, productivity loss, intangible costs to be captured, and data from community sectors and low-resource settings and countries.

## Introduction

Urinary tract infections (UTIs) are infections of the kidneys, bladder, or urethra defined by a combination of clinical features and the presence of bacteria in urine. These are some of the most common conditions managed in primary care, with approximately 75% of women experiencing at least one episode in their lifetime [1]. Consequently, UTIs are the second most common reason for primary care antibiotic prescribing in England [2,3]. However, it is estimated that up to 50% of these prescriptions were inadequate [4,5]. If managed inappropriately, in cases such as undertreating, subsequent sequelae include recurrent infections, bacteremia, sepsis, and potential mortality [2]. In addition, inappropriate management of UTIs, including overusing antibiotics (ie, using antibiotics when not required or for prolonged durations), accelerates the emergence and transmission of antibiotic resistance (ABR) in the long-term [6]. An increasing level of ABR in the community poses challenges to infection due to the higher risk of first-line antibiotic regime failure [7]. In the United Kingdom, the susceptibility of *Escherichia coli’s* (*E coli*), the most common cause of UTIs, to first-line treatments of trimethoprim and nitrofurantoin is declining [8]. This may have resulted in a rise in bacteremia caused by drug-resistant Gram-negative bacteria (GNB), as over 40% of *E coli* bacteremia had a urinary source [9].

Drug-resistant UTIs impose an economic burden on individuals, health care systems, and society as a whole [10-13]. The reduced effectiveness of UTI antibiotics can lead to repeated and more extensive treatment, hospital admission and prolonged length of stay (LOS), increased medical costs, and mortality [14]. The UK government has set new commitments in the National Action Plan to improve the prevention and control of UTIs in the community, particularly for older adults, and to gain a better understanding of the economic impacts of ABR [15]. Despite the high prevalence of UTIs in the community, evidence of the financial and human costs associated with drug-resistant UTIs is scarce, particularly due to the difficulties in quantifying costs incurred outside secondary care [11]. An understanding of the clinical and economic burden of antibiotic-resistant UTIs is key to evaluating the cost-effectiveness of stewardship interventions, including those aimed at using point-of-care diagnosis, clinical decision support tools, and reducing prescribing in the community [16]. In this research, we sought to systematically synthesize the evidence on the economic burden associated with antibiotic-resistant community-acquired UTIs (CA-UTIs), using published studies comparing the costs of antibiotic-susceptible and antibiotic-resistant cases.

## Methods

This systematic review followed the PRISMA (Preferred Reporting Items for Systematic Reviews and Meta-Analyses) guidance [17] and was registered at PROSPERO (CRD42023374551).

### Search Methods

We searched for studies estimating the economic costs attributable to antibiotic drug-resistant CA-UTIs published from January 1, 2008, to January 31, 2023, using a combination of broad-based (and wildcard) search criteria, including terms for UTI, community-acquired, ABR, and health economic cost. We searched the PubMed, Ovid MEDLINE and Embase, Cochrane Review Library, and Scopus databases using strings developed for each database (Table S2 in Multimedia Appendix 1). The bibliographies of the identified studies were also reviewed.

### Study Selection

The study inclusion/exclusion criteria are presented in Table 1, including the Patient/Population, Intervention, Comparison, and Outcomes (PICO) eligibility. Two authors (NJZ and MW) independently screened the titles and abstracts of the records yielded from the database search and independently screened the full-text articles. The discrepancies during title/abstract screening and full-text screening were resolved by consulting the third author (EC). Any article comparing monetary or health economic costs of antibiotic-resistant versus susceptible CA-UTIs through clinical trials, observational designs (eg, cohort study, case-control study), or modeling approaches was included for full-text review.

**Table 1.** Study inclusion/exclusion criteria.

Criteria		Inclusion	Exclusion
Article type		Clinical trials Observational designs (eg, cohort study, case-control study) Modeling approach (eg, economic evaluation)	Abstracts without full text Studies with small samples (eg, case reports) Studies with no primary evidence (eg, reviews, commentaries, editorials, or letters)
Language		English	Other languages
**PICO^a^ eligibility**			
	Population	Humans All ages All sexes Patients with community-acquired urinary tract infections	Animals Environmental studies Patients with health care–associated urinary tract infections Patients with infections from other locations
	Intervention/exposure	Infected by antibiotic-susceptible bacteria Infected by antifungal-susceptible fungi	Infected by virus Infected parasites
	Comparison/control	Infected by antibiotic nonsusceptible/resistant bacteria Infected by antifungal nonsusceptible/resistant fungi	Infected by virus Infected parasites
	Outcomes	Mortality Hospital length of stay Direct and indirect medical costs	Other outcomes (eg, patient satisfaction)

a PICO: Patient/Population, Intervention, Comparison, and Outcomes.

### Data Extraction and Analysis

Data were extracted from the included studies, including study identifier, authors, journal, publication year, study design, data collection period, country/region, health care setting, perspective (patient, health system [representing payer or provider], or societal), patient population, number of patients, UTI diagnosis criteria, pathogen, sensitivity profile, treatment, and outcome. We synthesized the impact of ABR on health outcomes (eg, mortality), health care system (eg, hospital LOS, medication cost), and economic system (eg, productivity), and compared these for infections caused by resistant versus susceptible pathogens. The methods to estimate the cost of illness were categorized using a top-down approach for those studies that reported total costs on a population level irrespective of the specific method used to derive these costs or a bottom-up approach for those studies that reported average costs derived from accumulating measured costs from patient samples.

A meta-analysis was performed to synthesize the reported mortality and hospital LOS using a random effect model [18]. A random effects model assumes that the true effect size of the exposure varies from study to study due to study heterogeneity. Particularly, heterogeneities in this type of analysis occurred in definitions and categories of costs across health systems, settings, and disease types; cost measurement instruments; and unit prices. Thus, a random effects model was chosen to allow aggregating cost data from different studies by circumventing this heterogeneity. In the meta-analysis of mortality, we estimated pooled odds ratios based on the crude mortality rate [19]. In the meta-analysis of LOS, we applied both the transformation-based methods (ie, estimating the sample mean and SD from the median and sample size) [20,21] and median-based methods (ie, considering study-specific median differences and data distribution) [22], considering mean and variance and median and IQR were commonly used when reporting LOS, and the distribution of LOS was heavily right-tailed (eg, not normally distributed) [23,24]. We assessed the publication bias for the mortality outcome using a funnel plot and Egger test [25,26]. No meta-analysis was performed for economic costs due to the large variation in the resource costs and the methods used to determine the cost. To compare the reported monetary costs, the outcomes were converted into 2023 US dollars by inflating the cost to 2023 original currency estimates using annual inflation rates [27], then converting this into US dollars utilizing the 2023 average exchange rates [28].

### Quality Assessment

The included studies were assessed using the Integrated Quality Criteria for Review of Multiple Study Designs (ICROMS) tool [29].

## Results

### Study Characteristics

**Figure 1.**
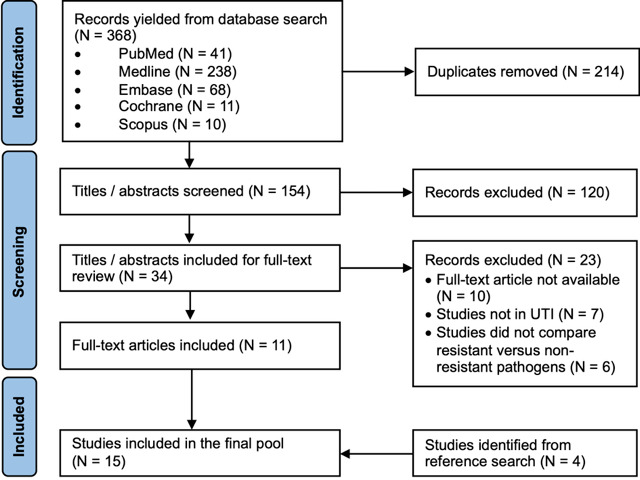
PRISMA (Preferred Reporting Items for Systematic Reviews and Meta-Analyses) flowchart. UTI: urinary tract infection.

A total of 380 titles and abstracts were yielded from the database search; 214 duplicates were removed, and 132 abstracts were deemed irrelevant. A full-text review was performed on 34 studies, of which, 11 studies were included. Through reference search, another 4 studies were identified and included in the final study pool. Figure 1 summarizes the screening process in a PRISMA flowchart.

The characteristics of the 15 identified studies are presented in Table 2 [12-14,30-41]. The countries that individually produced the highest number of studies were the United States (n=5, 33%) [14,33,38,39,41], followed by Spain (n=3, 20%) [13,32,40] and South Korea (n=2, 13%) [30,36]. A total of 13 (87%) studies focused on patients who were hospitalized [12-14,30,32-36,38-41], and 2 (13%) studies focused on primary care patients [31,37]. Additionally, 13 (87%) studies included adult patients of all genders [12-14,32-41], of which, 1 study included patients 65 years and older [32]. Chang et al [30] and Little et al [31] (n=2, 13%) investigated adult female patients. All hospital-based studies had UTI diagnosed via the presence of symptoms, infection biomarkers, and microbiology culture confirmation, and differentiated community-acquired cases using the 48-hour cutoff time after admission. Two (13%) studies reported hospital-acquired UTI [14,39]. The community-based study recruited patients with urinary tract symptoms (suspected UTI) or a history of dysuria and frequency [31,37]. In total, 57,251 CA-UTI cases were reported, and 47,131 UTI cases were analyzed (Table S3 in Multimedia Appendix 1).

**Table 2.** Study characteristics: data collection period, patient population, and identified pathogens.

Study	Country	Period	Population	Organisms identified		
				Gram-negative	Gram-positive	Fungi
Chang et al [30], 2016	South Korea	January 2001-December 2010	Hospitalized female patients with CO^a^-APN^b^ defined by presence of fever (≥38.0 °C), pyuria (5‐10 leukocytes per HPF^c^ upon urine microscopic examination), bacteriuria (≥105/ml clean voided urine or ≥104/ml catheterized urine)	*Escherichia coli*	—^d^	—
Sozen et al [12], 2015	Turkey	July 2012-June 2014	Hospitalized patients with positive urine culture <48 hours after admission, without hospitalization or urological surgery during the last month	*Enterobacter aerogenes* *Escherichia coli* *Klebsiella pneumoniae* *Pseudomonas aeruginosa*	—	—
Little et al [31], 2009	UK	April 2002-May 2003	Female patients aged 17‐70 years recruited from primary care practices with suspected UTI^e^ or a history of dysuria and frequency	Not reported	Not reported	Not reported
Tabak et al [14], 2018	US	January 2013-September 2015	Hospitalized adult patients with urine culture <3 days after admission, with Gram-negative pathogens isolated and tested for carbapenem susceptibility	*Acinetobacter baumannii* *Citrobacter freundii* *Enterobacter aerogenes* *Enterobacter cloacae* *Escherichia coli* *Klebsiella pneumoniae* *Morganella morganii* *Proteus mirabilis* *Pseudomonas aeruginosa* *Serratia marcescens*	—	—
Madrazo et al [32], 2021	Spain	January 2016-December 2019	Hospitalized patients aged ≥65 years with CA^f^-UTI and positive urine culture	*Acinetobacter baumannii* *Enterobacter cloacae* *Escherichia coli* *Klebsiella oxytoca* *Klebsiella pneumoniae* *Proteus mirabilis* *Pseudomonas aeruginosa* *Other Enterobacterales*	*Enterococcus faecalis* *Enterococcus faecium* *Enterococcus gallinarum* *Streptococcus agalactiae*	*Candida* spp
Wozniak et al [34], 2022	Australia	January 2012-September 2016	Hospitalized patients with positive urine culture <48 hours after admission with >2 species identified (>105 CFUs^g^/ml, 103/ml for cystitis, 104/ml for pyelonephritis)	*Escherichia coli* *Klebsiella pneumoniae* *Pseudomonas aeruginosa*	*Enterococcus faecium* *Staphylococcus aureus*	
Zilberberg et al [41], 2017	US	2009-2013	Hospitalized adult patients aged ≥18 years with CO-UTI defined by *ICD-9*^h^ code, positive urine culture, and antibiotic treatment beginning <48 hours after admission and continuing for at least 3 consecutive days or until discharge	*Citrobacter freundii* *Escherichia coli* *Enterobacter aerogenes* *Enterobacter cloacae* *Klebsiella oxytoca* *Klebsiella pneumoniae* *Morganella morganii* *Serratia marcescens* *Proteus mirabilis* *Proteus* spp *Providencia* spp	—	—
Mark et al [33], 2021	US	January 2017-June 2019	Hospitalized patients aged ≥18 years with febrile UTI defined by fever, *ICD-10*^i^ code of UTI, pyelonephritis, or sepsis, urine culture (EKP species >100,000 CFUs/ml)	*Escherichia coli* *Klebsiella pneumoniae* *Proteus mirabilis*	—	—
Kim et al [36], 2013	South Korea	March 2010-February 2011	Hospitalized patients admitting emergency department or outpatient clinic from the community with CA-APN defined by pyuria (≥5‐9 WBC^j^/HPF), fever (≥37.8 °C), and positive urine culture collected at the time of admission	*Acinetobacter baumannii* *Citrobacter* spp *Enterobacter* spp *Escherichia coli* *Klebsiella pneumoniae* *Proteus* spp *Pseudomonas aeruginosa*	*Enterococcus* spp *Staphylococcus aureus*	—
François et al [37], 2016	France	January 2012-February 2013	Female patients aged >18 years recruited from GPs^k^ with UTI symptoms and followed up for 8 weeks	*Escherichia coli*	—	—
Cheong et al [35], 2022	Korea	January 2018-December 2019	Hospitalized patients aged ≥19 years with *ICD-10* code of CA-APN <48 hours after admission, defined by fever (≥37.8 °C), pyuria (≥4‐9 WBC/HPF), positive urine or blood culture, and symptoms or signs relevant to APN	*Citrobacter* spp *Enterobacter* spp *Escherichia coli* *Klebsiella pneumoniae* *Proteus* spp	—	—
MacVane et al [38], 2013	US	September 2011-August 2012	Hospitalized patients aged ≥18 years with UTI present ≤48 hours after admission defined by positive urine culture (≥10,000 CFUs)	*Escherichia coli* *Klebsiella* spp	—	—
Esteve-Palau et al [13], 2015	Spain	August 2010-July 2013	Hospitalized patients aged ≥18 years with symptomatic CA- or CO-HA^l^-UTI ≤48 hours after admission including cystitis, pyelonephritis, acute prostatitis, and urosepsis, defined by increases in urinary frequency, urgency, dysuria, or suprapubic tenderness, a positive urine culture of *Escherichia coli* (>105 CFUs/ml)	*Escherichia coli*	—	—
Rozenkiewicz et al [40], 2021	Spain	January 2011-January 2016	Hospitalized patients aged ≥18 years with symptomatic CA-UTI (identified ≤48 hours after admission and not AHA^m^) including cystitis, pyelonephritis, acute prostatitis, urinary sepsis, and confusion state associated with UTI, defined by fever (>38 °C), urinary urgency, polyuria, dysuria or suprapubic pain, a positive urine culture (>105 CFUs/ml)	*Klebsiella pneumoniae*	—	—
Cardwell et al [39], 2016	US	July 2013-September 2013	Hospitalized patients aged ≥18 years with fever, chills, rigors, nausea, or vomiting; hematuria; altered mental status; suprapubic or flank pain; costovertebral angle tenderness; urinary frequency, urgency, or dysuria; and treatment for UTI ≤24 hours after admission	*Citrobacter* spp *Enterobacter* spp *Escherichia coli* *Klebsiella* spp *Morganella* spp *Proteus* spp *Providencia* spp *Pseudomonas aeruginosa* *Serratia* spp	*Enterococcus* spp	—

a CO: community-onset.

b APN: acute pyelonephritis.

c HPF: high-power field.

d Not applicable.

e UTI: urinary tract infection.

f CA: community-acquired.

g CFU: colony-forming unit.

h *ICD-9*: *International Classification of Diseases, Ninth Revision*.

i *ICD-10*: *International Statistical Classification of Diseases, Tenth Revision*.

j WBC: white blood cell.

k GP: general practice.

l HA: hospital-acquired.

m AHA: ambulatory health care associated.

Of the 15 studies, 14 (93%) reported the pathogens identified, of which, all reported GNB [12-14,30-41], 4 (29%) reported Gram-positive bacteria [32,34,36,39], 1 (7%) reported fungi [32], 3 (21%) exclusively reported UTI caused by *E coli* [13,30,37], 1 (7%) reported UTI caused by *K pneumoniae* [40]. *E coli*, *K pneumoniae*, and *P aeruginosa* are the most frequently identified organisms. Among the studies in specific antibiotic-pathogen combinations, 2 studies assessed carbapenem-resistant organisms, specifically GNB and Enterobacterales [14,41]. Mark et al [33] examined *E coli*, *K pneumoniae*, and *Proteus mirabilis* (*P mirabilis*) resistance to third-generation cephalosporins. Sozen et al [12] and MacVane et al [38] examined extended-spectrum β-lactamases– or inducible β-lactamases–producing GNB.

All the included studies estimated the clinical and economic outcomes of patients recruited from single or multiple health facilities. François et al [37] provided a national-level estimate of the infection incidence and costs derived from the study cohort. No study performed sensitivity analysis. The results of the quality assessment are presented in Table S5 in Multimedia Appendix 1. All studies met the minimum required score. Of the 15 studies, 6 (40%) failed to meet the minimum required criteria [13,30,37,38,40,41].

### The Burden of ABR UTIs

When quantifying the burden attributable to ABR, the included studies compared patient outcomes, health system outcomes, and economic costs of the CA-UTI cases caused by resistant pathogens against those caused by nonresistant pathogens. The most reported outcomes were mortality, hospital LOS, and economic costs due to antibiotic treatment (Table S4 in Multimedia Appendix 1). A health system perspective was taken by all except 1 study when estimating the costs [12-14,30-41]. François et al [37] took a societal perspective and included productivity loss due to absenteeism. When comparing the patients with resistant and nonresistant CA-UTIs, 4 studies matched case and control [13,14,38,41], 2 studies adjusted patient characteristics and other risk factors when reporting outcomes [33,35], other studies performed no matching or adjusting.

**Figure 2.**
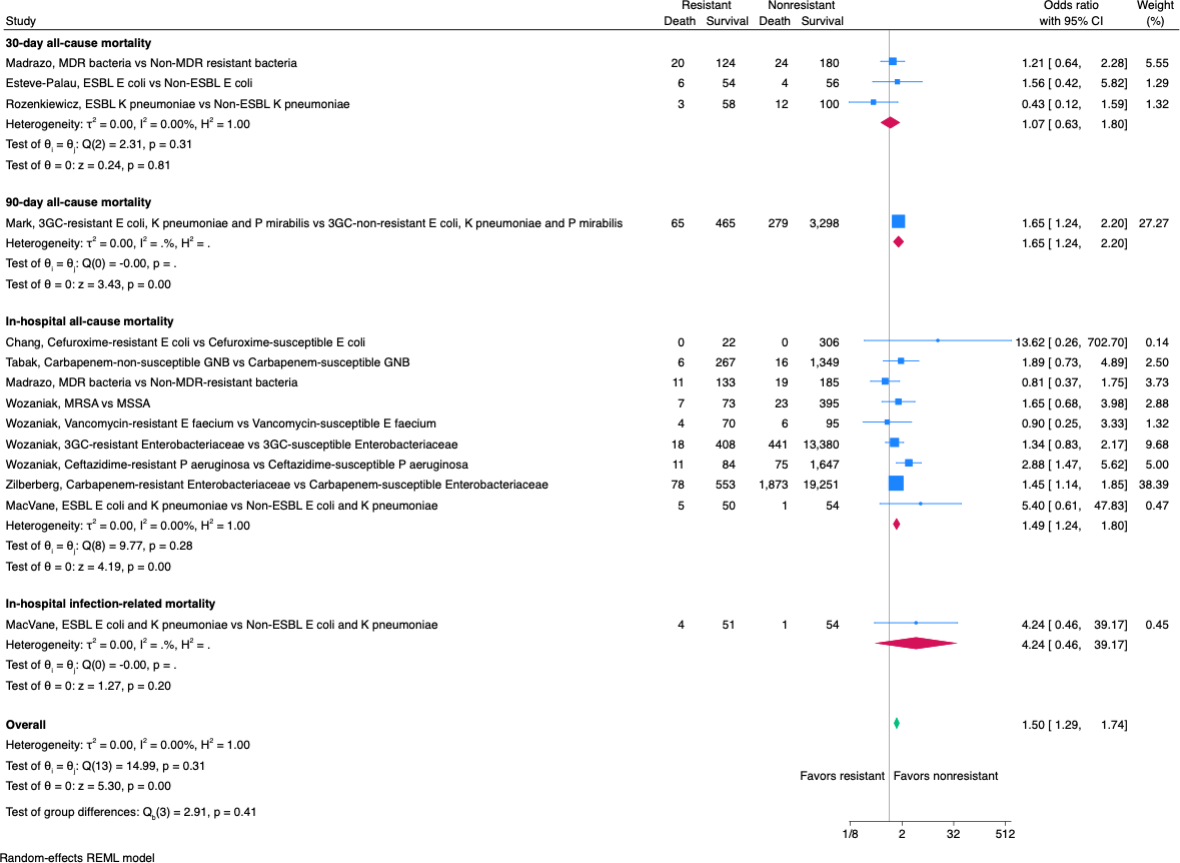
Pooled mortality of urinary tract infections [13,14,30,32-34,38,40,41]. 3GC: third-generation cephalosporin-resistant; ESBL: extended-spectrum β-lactamase; GNB: Gram-negative bacteria; MDR: multidrug resistant; MRSA: methicillin-resistant *Staphylococcus aureus*; MSSA: methicillin-sensitive *Staphylococcus aureus*.

A total of 12 studies reported mortality, including in-hospital all-cause mortality [14,30,32,34,38,41], in-hospital infection-related mortality [38], 30-day all-cause mortality [13,32,40], and 90-day all-cause mortality [33] (Table S4 A in Multimedia Appendix 1). A total of 7 studies reported higher crude mortality among the patients with antibiotic-resistant UTIs [13,14,32-34,38,41], of which, 1 study demonstrated the statistical significance [41]. The pooled odds ratios of mortality outcomes for resistant UTIs are presented in Figure 2. Results presented odds ratios of resistant compared to nonresistant infections. The blue squares centered at the point estimate the effect size, with horizontal lines depicting the 95% CIs, and the sizes of the blue squares correspond to the patient group sizes. The overall effect sizes are represented by diamonds centered on their estimated values with the diamond width corresponding to the CI length. The random effects model estimated an overall odds ratio of 1.50 (95% CI 1.29-1.74), suggesting that ABR increased the overall mortality. The subgroup analysis conducted for different mortality outcomes suggested increased odds of in-hospital all-cause mortality (Figure 2). No publication bias was detected for mortality (Figure S1 in Multimedia Appendix 1).

**Figure 3.**
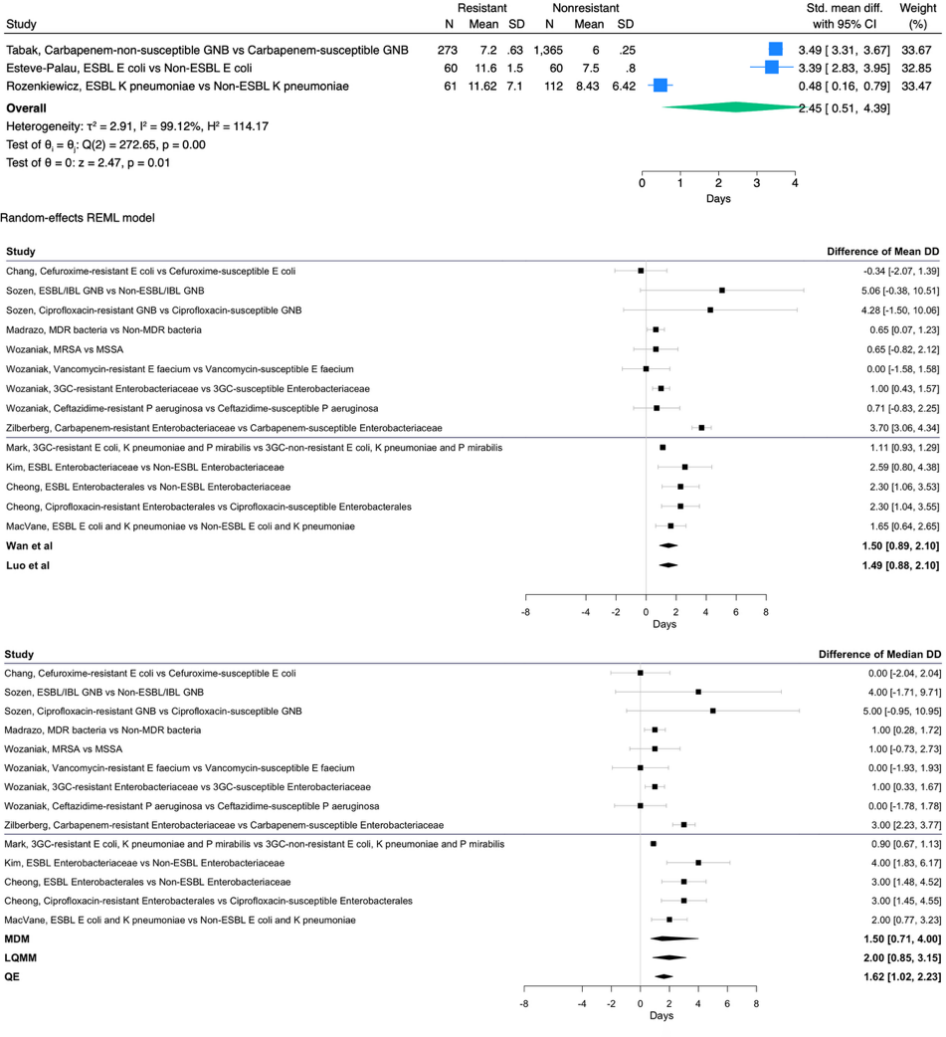
(A) Pooled mean difference in length of stay of urinary tract infections. (B) Pooled median difference in length of stay of urinary tract infections [12-14,20,21,30,32-36,38,40,41]. 3GC: third-generation cephalosporin-resistant; ESBL: extended-spectrum β-lactamase; GNB: Gram-negative bacteria; IBL: inducible β-lactamase; LQMM: linear quantile mixed model; MDM: median of the differences of medians; MDR: multidrug resistant; MRSA: methicillin-resistant *Staphylococcus aureus*; MSSA: methicillin-sensitive *Staphylococcus aureus*; QE: test for residual heterogeneity; REML: restricted or residual maximum likelihood.

All 13 hospital-based studies reported LOS [12-14,30,32-36,38-41], among which, 11 reported significantly higher LOSs associated with antibiotic-resistant UTIs (Table S4 B in Multimedia Appendix 1) [12-14,32-36,38,40,41]. Cardwell et al [39] reported higher LOS among patients with clinical failure due to inappropriate antibiotic therapies for resistant infections. The meta-analysis of studies reported LOS in mean and SD estimates of a pooled excess LOS of 2.45 days (95% CI 0.51‐4.39; Figure 3A). The meta-analysis of studies reported LOS in median and IQR estimates of a pooled excess LOS, ranging from the lowest value of 1.50 days (95% CI 0.71-4.00), estimated by the median of the differences of medians method, to the highest value of 2.00 days (95% CI 0.85-3.15), estimated by the linear quantile mixed models method (Figure 3A).

**Figure 4.**
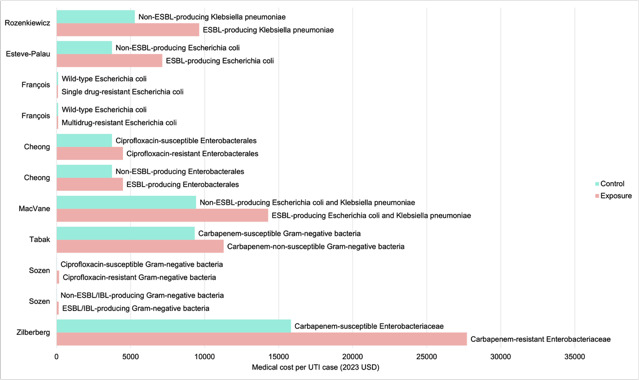
Medical cost of antibiotic-resistant urinary tract infections [12-14,35,37,38,40,41]. ESBL: extended-spectrum β-lactamase; IBL: inducible β-lactamase; UTI: uniary tract infection.

A total of 8 studies reported costs in monetary terms (Table S4 C in Multimedia Appendix 1) [12-14,35,37,38,40,41], including 5 that reported costs in US dollars [12,14,35,38,41] and 3 that reported costs in euros [13,37,40] (Figure 4). None of the included studies discounted the costs. Considering only 2 studies explicitly stated the year of which the costs were adjusted to [12,37], the end year of the data collection period was used to convert the reported costs into 2023 US dollars. A total of 8 studies reported direct medical costs incurred in secondary care, including emergency department costs [13,33] and outpatient parenteral antibiotic therapy costs in 1 study [13,33]. All 8 studies reported higher medical costs spent treating patients with resistant UTIs in hospitals. The highest excess cost was observed in UTIs caused by carbapenem-resistant Enterobacterales [41]. François et al [37] reported costs incurred in primary care, specifically, the costs of GP visits due to UTI symptoms. The primary care costs of single- or multidrug-resistant *E coli* UTIs were not significantly higher than those caused by susceptible *E coli*.

## Discussion

This review concluded that there is an economic burden attributable to ABR in CA-UTIs, including the costs for patients and health systems as well as costs at the societal level. The review included 15 studies, which were overrepresented by research from high-income countries, hospital settings, and infections caused by *E coli* and *K pneumoniae*. All studies were cross-sectional with a limited patient sample size. No sensitivity analysis was performed to quantify the level of uncertainty in the results. The meta-analysis provided pooled estimates of the odds ratio of mortality and mean differences in hospital LOS. The reported variation in economic costs was also synthesized.

We found that no systematic review on the economic burden of ABR in CA-UTIs had been conducted. The increased mortality among the patients with ABR CA-UTIs in this review was less profound, as opposed to the existing research in other types of infections, such as bacteremia [40-42] or health care–associated UTIs [43]. Overall, ABR is attributed to an increased mortality odds ratio of 1.50. The increased odds of mortality can be explained by the higher risk of treatment failure and UTI complications such as bacteremia and sepsis. The varied types of mortality outcomes reported reduced the comparability across studies. Most of the hospital-based studies reported a longer LOS experienced by the patients in the ABR group. We used multiple modeling methods for the hospital LOS meta-analysis and estimated that the excess duration of hospitalization ranged from 1.50 to 2.45 days. All the studies that captured the costs in monetary terms reported excess medical costs in the ABR group, with the highest excess medical costs being US $11,884.32 per case of CA-UTI caused by carbapenem-resistant Enterobacterales [42,43]. The findings of this review highlighted the scarcity of research in quantifying the economic burden of ABR, particularly in four areas. First, besides mortality, evidence of other types of patient burden associated with ABR is lacking, such as morbidity (clinical failure, time to clinical stability, secondary infections) and chronic sequelae (recurrent infections). Second, existing research has been restricted to those cases present in the hospitals; the cases managed and the costs incurred in primary care settings were not captured. However, the pathogen distributions and treatment options varied substantially for hospital-acquired and CA-UTIs, and for CA-UTIs managed in the community and in hospitals; community-based investigation is urgently needed to generate a comprehensive understanding across the whole health economy [42,43]. Third, the types of medical resource costs remained largely inconsistent, which further reduced the validity of the excess costs estimated. Last, all the identified studies were limited in patient cohort size and follow-up duration and lacked analysis to address uncertainty, which led to concerns about the results’ generalizability.

This review has two limitations. First, we only searched for studies published in English. Second, we did not include those studies where the primary focus was to perform an economic evaluation of CA-UTI treatment or prevention measures and the included estimated costs of drug-resistant cases. These limitations provide scope for further research.

There is a pressing need to build an understanding of the economics of AMR. The evidence to provide a full economic case for interventions tackling AMR is lacking. In this review, we identified knowledge and methodological gaps in existing research particularly relevant to quantifying costs associated with ABR that occurred in the community. Future research calls for cost-of-illness analysis of infections standardizing therapy-pathogen combination comparators, medical resources, productivity loss, and intangible costs to be captured, as well as data from community sectors and low-resource settings and countries.

## Supplementary material

10.2196/53828 Multimedia Appendix 1 Supplementary data tables.

10.2196/53828 Checklist 1 PRISMA (Preferred Reporting Items for Systematic Reviews and Meta-Analyses) checklist.

## Abbreviations

ABR: antibiotic resistance
CA-UTI: community-acquired urinary tract infection
GNB: Gram-negative bacteria
ICROMS: Integrated Quality Criteria for Review of Multiple Study Designs
LOS: length of stay
PICO: Patient/Population, Intervention, Comparison, and Outcomes
PRISMA: Preferred Reporting Items for Systematic Reviews and Meta-Analyses
UTI: urinary tract infection
