# Supplementary material for: Economic Burden of Community-Acquired Antibiotic-Resistant Urinary Tract Infections: Systematic Review and Meta-Analysis
Source: JMIR Public Health Surveill. 2024 Oct 9;10:e53828. doi: 10.2196/53828 (PMC11481822; doi:10.2196/53828)
Supplement: Multimedia Appendix 1 [file publichealth-v10-e53828-s001.docx]

**Table S2 Database search strings**

| **Search terms** | | **Results** |
| --- | --- | --- |
| PubMed | | |
| 1 | "Community-acquired"[Title/Abstract] OR "Community-onset"[Title/Abstract] OR "general practice"[Title/Abstract] OR "primary care"[Title/Abstract] | 197,781 |
| 2 | "Urine"[Title/Abstract] OR "urinary tract infection"[Title/Abstract] OR "urethra infection"[Title/Abstract] OR "Urethritis"[Title/Abstract] OR "bladder infection"[Title/Abstract] OR "Cystitis"[Title/Abstract] OR "Cystalgia"[Title/Abstract] OR "Bacteriuria"[Title/Abstract] OR "Bacilluria"[Title/Abstract] OR "Pyuria"[Title/Abstract] OR "Dysuria"[Title/Abstract] OR "Pyelonephritis"[Title/Abstract] OR "Leukocyturia"[Title/Abstract] | 190,676 |
| 3 | "Resistant"[Title/Abstract] OR "Drug-resistant"[Title/Abstract] OR "multi drug resistant"[Title/Abstract] OR "antimicrobial resistant"[Title/Abstract] OR "antibiotic resistant"[Title/Abstract] OR "antibacterial resistant"[Title/Abstract] OR "non susceptible"[Title/Abstract] | 521,035 |
| 4 | “Cost” [Title/Abstract] OR “economic” [Title/Abstract] OR “burden” [Title/Abstract] OR “mortality” [Title/Abstract] OR “length of stay”[Title/Abstract] OR “length of hospitali*” [Title/Abstract] OR “duration of stay” [Title/Abstract] OR “duration of hospitali*” [Title/Abstract] OR “productivity”[Title/Abstract] | 1,989,673 |
| 5 | #1 AND #2 AND #3 AND #4 | 72 |
| 6 | Limit #5 to English only | 72 |
| 7 | Limit #6 to human only | 56 |
| 8 | Limit #7 to 2018/1/1 - 2023/1/31 | 48 |
| MEDLINE | | |
| 1 | ("Community-acquired" or "Community-onset" or "General practice" or "Primary care").at,ab. | 498,525 |
| 2 | (Urine or "Urinary tract infection" or Urethra or "infection Urethritis" or "Bladder infection" or Cystitis or Cystalgia or Bacteriuria or Bacilluria or Pyuria or Dysuria or Pyelonephritis or Leukocyturia).at,ab. | 873,465 |
| 3 | (Resistant or Drug-resistant or Multi-drug resistant or Antimicrobial resistant or Antibiotic resistant or Antibacterial resistant or Non susceptible).at,ab. | 1,378,867 |
| 4 | (Cost or economic or burden or mortality or “length of stay” or “length of hospitali*” or “duration of stay” or “duration of hospitali*” or productivity).at,ab. | 5,685,370 |
| 5 | 1 and 2 and 3 and 4 | 277 |
| 6 | Limit 5 to English language | 271 |
| 7 | Limit 6 to human | 256 |
| 8 | Limit 7 to dt=20120101-20230131 [January 1st, 2012 to January 31st, 2023] | 243 |
| Embase | | |
| 1 | ("Community-acquired" or "Community-onset" or "General practice" or "Primary care").at,ab. | 219,111 |
| 2 | (Urine or "Urinary tract infection" or Urethra or "infection Urethritis" or "Bladder infection" or Cystitis or Cystalgia or Bacteriuria or Bacilluria or Pyuria or Dysuria or Pyelonephritis or Leukocyturia).at,ab. | 453,850 |
| 3 | (Resistant or Drug-resistant or Multi-drug resistant or Antimicrobial resistant or Antibiotic resistant or Antibacterial resistant or Non susceptible).at,ab. | 641,820 |
| 4 | (cost or economic or burden or mortality or length of stay or length of hospitali* or duration of stay or duration of hospitali* or productivity).at,ab. | 2,720,943 |
| 5 | 1 and 2 and 3 and 4 | 123 |
| 6 | Limit 5 to English language | 119 |
| 7 | Limit 6 to human | 117 |
| 8 | Limit 7 to dd=20120101-20230131 [January 1st, 2012 to January 31st, 2023] | 68 |
| Cochrane | | |
| 1 | ("Community-acquired" or "Community-onset" or "General practice" or "Primary care"):ti,ab | 26,496 |
| 2 | (Urine or "Urinary tract infection" or Urethra or "infection Urethritis" or "Bladder infection" or Cystitis or Cystalgia or Bacteriuria or Bacilluria or Pyuria or Dysuria or Pyelonephritis or Leukocyturia):ti,ab | 40,746 |
| 3 | (Resistant or Drug-resistant or Multi-drug resistant or Antimicrobial resistant or Antibiotic resistant or Antibacterial resistant or Non susceptible):ti,ab | 26,112 |
| 4 | (cost or economic or burden or mortality or length of stay or length of hospitali* or duration of stay or duration of hospitali* or productivity):ti,ab | 190,072 |
| 5 | #1 and #2 and #3 and #4 | 15 |
| 6 | #5 with Cochrane Library publication date from Jan 2012 to Jan 2023 | 11 |
| Scopus | | |
| 1 | TITLE-ABS ("community-acquired" OR "community-onset" OR "general practice" OR "primary care") | 225,402 |
| 2 | TITLE-ABS (urine OR "Urinary tract infection" OR urethra OR "infection Urethritis" OR "Bladder infection" OR cystitis OR cystalgia OR bacteriuria OR bacilluria OR pyuria OR dysuria OR pyelonephritis OR leukocyturia ) | 421,005 |
| 3 | TITLE-ABS (resistant OR "Drug-resistant" OR "Multi-drug resistant" OR "Antimicrobial resistant" OR "Antibiotic resistant" OR "Antibacterial resistant" OR "Non susceptible" ) | 786,611 |
| 4 | TITLE-ABS (cost OR economic OR burden OR mortality OR length of stay OR length of hospitali* OR duration of stay OR duration of hospitali* OR productivity) | 34,989 |
| 5 | #1 AND #2 AND #3 AND #4 | 13 |
| 6 | Language (English) | 13 |
| 7 | ((PUBYEAR > 2012) AND (PUBYEAR < 2023)) | 10 |

**Table S3 Study characteristics: urinary tract infection criteria, incidence, and causative pathogens**

|  | **Study** | **UTI (n)** | **Further inclusion / exclusion criteria** | **UTI analysed (n)** | **Causative pathogens (n, %)** | | |
| --- | --- | --- | --- | --- | --- | --- | --- |
|  |  |  |  |  | Escherichia coli | Klebsiella species | Pseudomonas aeruginosa |
| 1 | Chang | 328 |  | 328 | 328 |  |  |
| 2 | Sozen | 82 |  | 82 | 69 | 10 | 3 |
| 3 | Little | 839 | With mid-stream urine specimen, symptoms and duration, and antibiotic resistance status available | 511 | Not reported | | |
| 4 | Tabak | 7171 | Propensity score matched carbapenem susceptible (C-S) and carbapenem non-susceptible (C-NS) cases | 1638 (273 C-NS vs 1365 C-S) | 335 | Not reported | 397 |
| 5 | Madrazo | 388 |  | 388 | 219 | 52 | 28 |
| 6 | Wozniak | 16,737 |  | 16,737 | 14,247 Enterobacteriaceae (Escherichia coli and Klebsiella spp) | | 1,817 |
| 7 | Zilberberg | 21,755 | Matched carbapenem-resistant Enterobacteriaceae (CRE) and carbapenem-susceptible Enterobacteriaceae (CSE) cases | 21,755 (631 CRE vs 21,124 CSE) | Not reported | | |
| 8 | Mark | 5922 | Caused by EKP pathogens (Escherichia coli, Klebsiella pneumoniae, and Proteus mirabilis) | 4113 | Not reported | | |
| 9 | Kim | 557 |  | 557 | 497 | 18 | 6 |
| 10 | François | 460 | Confirmed by positive culture | 345 | 267 | Not reported | |
| 11 | Cheong | 241 | Caused by Enterobacteriales | 241 | 217 (90.0%) | 13 (5.4%) | Not reported |
| 12 | MacVane | 2345 | Matched ESBL-producing and non-ESBL producing cases | 55 | 44 | 8 |  |
| 13 | Esteve-Palau | 120 |  | 120 | 120 |  |  |
| 14 | Rozenkiewicz | 173 |  | 173 |  | 173 |  |
| 15 | Cardwell | 133 | Confirmed by positive culture | 88 | 51 | 10 | Not reported |

**Table S4 A Reported mortality**

| **Study** | **Comparator** | | **Infection** | | **Mortality** | | **Crude mortality rate (%)** | | |
| --- | --- | --- | --- | --- | --- | --- | --- | --- | --- |
|  | Exposure | Control | Exposure  (n) | Control  (n) | Exposure  (n) | Control  (n) | Exposure  (%) | Control  (%) | p-value |
| In-hospital all-cause mortality | | | | | | | | |  |
| Chang | Cefuroxime-resistant E. coli | Cefuroxime-susceptible E. coli | 22 | 306 | 0 | 0 | 0.0% | 0.0% | - |
| Tabak | Carbapenem-non-susceptible GNB | Carbapenem-susceptible GNB | 273 | 1365 | 6 | 16 | 2.2% | 1.2% | - |
| Madrazo | MDR | Non-MDR | 144 | 204 | 11 | 19 | 7.6% | 9.3% | 0.699 |
| Wozaniak | MRSA | MSSA | 80 | 418 | 7 | 23 | 8.8% | 5.5% | - |
| Wozaniak | Vancomycin-resistant E. faecium | Vancomycin-susceptible E. faecium | 74 | 101 | 4 | 6 | 5.4% | 5.9% | - |
| Wozaniak | 3GC-resistant Enterobacteriaceae | 3GC-susceptible Enterobacteriaceae | 426 | 13,821 | 18 | 441 | 4.2% | 3.2% | - |
| Wozaniak | Ceftazidime-resistant P. aeruginosa | Ceftazidime-susceptible P. aeruginosa | 95 | 1,722 | 11 | 75 | 11.6% | 4.4% | - |
| Zilberberg | Carbapenem-resistant Enterobacteriaceae | Carbapenem-susceptible Enterobacteriaceae | 631 | 21,124 | 78 | 1,873 | 12.4% | 8.9% | 0.002 |
| MacVane | ESBL-producing EK | Non-ESBL-producing EK | 55 | 55 | 5 | 1 | 9.1% | 1.8% | 0.21 |
| In-hospital infection-related mortality | | | | | | | | |  |
| MacVane | ESBL-producing EK | Non-ESBL-producing EK | 55 | 55 | 4 | 1 | 7.3% | 1.8% | 0.37 |
| 30-day all-cause mortality | | | | | | | | |  |
| Madrazo | MDR | Non-MDR | 144 | 204 | 20 | 24 | 13.9% | 11.8% | 0.624 |
| Esteve-Palau | ESBL-producing E. coli* | Non-ESBL-producing E. coli | 60 | 60 | 6 | 4 | 10.0% | 6.7% | 0.74 |
| Rozenkiewicz | ESBL-producing K. pneumoniae* | Non-ESBL-producing K. pneumoniae | 61 | 112 | 3 | 12 | 4.9% | 10.7% | 0.263 |
| 90-day all-cause mortality | | | | | | | | |  |
| Mark | 3GC-resistant EKP | 3GC-non-resistant EKP | 530 | 3,577 | 65 | 279 | 12.3% | 7.8% | - |
| *Reported with HCA-UTI and CA-UTI combined | | | | | | | | | |
| **Abbreviations:** GNB: Gram-negative bacteria; MDR: multi-drug resistant; MRSA: Methicillin-resistant Staphylococcus aureus; MSSA: Methicillin-susceptible Staphylococcus aureus; 3GC: third-generation cephalosporin; ESBL: Extended Spectrum Beta-Lactamase; E. coli: Escherichia coli; E. faecium: Enterococcus faecium; P. aeruginosa: Pseudomonas aeruginosa; K. pneumoniae: Klebsiella pneumoniae; EK: Escherichia coli and Klebsiella species; EKP: Escherichia coli, Klebsiella pneumoniae, and Proteus mirabilis. | | | | | | | | | |

**Table S4 B Reported hospital length of stay (LOS)**

| **Study** | **Comparator** | | **Infection** | | **Hospital LOS** | | |
| --- | --- | --- | --- | --- | --- | --- | --- |
|  | Exposure | Control | Exposure  (n) | Control  (n) | Exposure  (days) (median, IQR) | Control  (days) (median, IQR) | p-value |
| Chang | Cefuroxime-resistant E. coli | Cefuroxime-susceptible E. coli | 22 | 306 | 10 (8 - 13) | 10 (8 - 14) | 0.319 |
| Sozen | ESBL/IBL positive GNB | ESBL/IBL negative GNB | 45 | 43 | 9 (3 - 24) | 5 (2 - 14) | 0.001 |
| Sozen | Ciprofloxacin-resistant GNB | Ciprofloxacin-susceptible GNB | 48 | 40 | 9 (3 - 24) | 4 (2 - 17) | 0.001 |
| Tabak | Carbapenem-non-susceptible GNB | Carbapenem-susceptible GNB | 273 | 1365 | Mean: 7.2 | Mean: 6.0 | < 0.001 |
| Madrazo | MDR | Non-MDR | 144 | 208 | 6 (4 - 8) | 5 (4 - 7) | 0.029 |
| Wozaniak | MRSA | MSSA | 80 | 418 | 6.5 (4 - 12.3) | 6 (3 - 12) | - |
| Wozaniak | Vancomycin-resistant E. faecium | Vancomycin-susceptible E. faecium | 74 | 101 | 6.5 (4 - 10.7) | 7 (4 - 11) | - |
| Wozaniak | 3GC-resistant Enterobacteriaceae | 3GC-susceptible Enterobacteriaceae | 426 | 13,821 | 6 (3 - 11) | 5 (3 - 9) | - |
| Wozaniak | Ceftazidime-resistant P. aeruginosa | Ceftazidime-susceptible P. aeruginosa | 95 | 1,722 | 6 (3 - 12.5) | 6 (3 - 11) | - |
| Zilberberg | Carbapenem-resistant Enterobacteriaceae | Carbapenem-susceptible Enterobacteriaceae | 631 | 21,124 | 10 (6 - 17)  Mean (SD): 14.6 (15.9) | 7 (4 - 11)  Mean (SD): 9.0 (9.4) | < 0.001 |
| Mark | 3GC-resistant EKP | 3GC-non-resistant EKP | 530 | 3,577 | 88.8h (64.8 - 132.0)  Mean (SD): 115.4 (117.8) | 67.2h (45.6 - 93.6)  Mean (SD): 87.1 (98.6) | - |
| Kim | ESBL-producing Enterobacteriaceae | Non-ESBL-producing Enterobacteriaceae | 46 | 480 | 10.5 (5.8 - 14.3) | 7 (6 - 10) | 0.012 |
| Cheong | ESBL-producing Enterobacterales | Non-ESBL-producing Enterobacterales | 75 | 166 | 11 (8 - 14) | 8 (6 - 12) | < 0.001 |
| Cheong | Ciprofloxacin-resistant Enterobacterales | Ciprofloxacin-susceptible Enterobacterales | 87 | 154 | 11 (7 - 14) | 8 (6 - 11) | < 0.001 |
| MacVane | ESBL-producing EK | Non-ESBL-producing EK | 55 | 55 | 6 (4 - 8) | 4 (3 - 6) | 0.02 |
| Esteve-Palau | ESBL-producing E. coli** | Non-ESBL-producing E. coli | 60 | 60 | Mean (SD): 11.6 (1.5) | Mean (SD): 7.5 (0.8) | 0.02 |
| Rozenkiewicz | ESBL-producing K. pneumoniae** | Non-ESBL-producing K. pneumoniae | 61 | 112 | Mean (SD): 11.62 (7.1) | Mean (SD): 8.43 (6.42) | 0.003 |
| Cardwell | CA-UTI with appropriate empirical therapy | CA-UTI with inappropriate empirical therapy | Not reported | Not reported | 4 (3-6)*** | 3 (4-10)*** | 0.79 |
| **Reported with HCA-UTI and CA-UTI combined | | | | | | | |
| **Abbreviations:** GNB: Gram-negative bacteria; MDR: multi-drug resistant; MRSA: Methicillin-resistant Staphylococcus aureus; MSSA: Methicillin-susceptible Staphylococcus aureus; 3GC: third-generation cephalosporin; ESBL: Extended Spectrum Beta-Lactamase; E. coli: Escherichia coli; E. faecium: Enterococcus faecium; P. aeruginosa: Pseudomonas aeruginosa; K. pneumoniae: Klebsiella pneumoniae; EK: Escherichia coli and Klebsiella species; CA-UTI: community-acquired urinary tract infection. | | | | | | | |

**Table S4 C Reported economic costs**

| **Study** | **Comparator** | | **Infection** | | **Costed items** | **Cost** | | | **Year to convert from** |
| --- | --- | --- | --- | --- | --- | --- | --- | --- | --- |
|  | Exposure | Control | Exposure  (n) | Control  (n) |  | Exposure  (mean, 95 % CI) | Control  (mean, 95 % CI) | p-value |  |
| Sozen | ESBL/IBL positive GNB | ESBL/IBL negative GNB | 45 | 43 | Antibiotic treatment | Median (IQR): $110.6 (5.5-505.2) | Median (IQR): $19.8 (6.5-384.2) | 0.001 | 2014 |
| Sozen | Ciprofloxacin-resistant GNB | Ciprofloxacin-susceptible GNB | 48 | 40 | Antibiotic treatment | Median (IQR): $135.1 (5.5 - 505.2) | Median (IQR): $19.8 (6.56 - 234.6) | 0.001 | 2014 |
| Tabak | Carbapenem-non-susceptible GNB | Carbapenem-susceptible GNB | 273 | 1,365 | Unspecified | $8,743 | $7,231, p < 0.001 | < 0.001 | 2015 |
| Zilberberg | Carbapenem-resistant Enterobacteriaceae | Carbapenem-susceptible Enterobacteriaceae | 631 | 21,124 | Unspecified | $33,400 (SD: 37,662)  Median (IQR): 21,154 (12,687 - 39,374, p < 0.001) | $19,036 (SD: 24,494)  Median (IQR): 12,082 (7,104 - 21,822, p < 0.001) | < 0.001 | 2013 |
| Cheong | ESBL-producing Enterobacterales | Non-ESBL-producing Enterobacterales | 75 | 166 | Consultation fee  Hospitalisation expenditures  Meal  Cost for medication  Procedure or operation  Laboratory examination  Radiologic examination  Others | Median (IQR): $3,730.2 (2,928.9 - 5,692.4) | Median (IQR): $3,119.3 (2,099.3 - 4,829.9) | 0.001 | 2019 |
| Cheong | Ciprofloxacin-resistant Enterobacterales | Ciprofloxacin-susceptible Enterobacterales | 87 | 154 |  | Median (IQR): $3,730.2 (2,524.4 - 5,937.7) | Median (IQR): $3,119.3 (2,148.3 - 4,578.5) | 0.005 | 2019 |
| MacVane | ESBL-producing EK | Non-ESBL-producing EK | 55 | 55 | Bed cost  Antibiotic treatment | Median (IQR): $10,741 (6,846 - 15,819) | Median (IQR): $7,083 (5,667 - 11,652) | 0.02 | 2012 |
| François | MDR E. coli* | Wild (susceptible) E. coli* | 98,504 | 222,933 | Physician visits  Diagnostic tests  Prescription drugs  Hospitalizations  Loss of productivity due to absenteeism | €74.49 (30.87 - 118.11) | €74.76 (57.61 - 91.91) | 0.99 | 2013 |
| François | SDR E. coli* | Wild (susceptible) E. coli* | 197,009 | 222,933 |  | €67.44 (43.93 - 90.95) | €74.76 (57.61 - 91.9) | 0.63 | 2013 |
| Esteve-Palau | ESBL-producing E. coli** | Non-ESBL-producing E. coli | 60 | 60 | Hospitalisation cost   - Pharmacy - Antibiotic treatment - Laboratory - Inter consultations   OPAT | Median (IQR): €4,980 (2,783 - 8,465) | Median (IQR): €2,612 (1,810 - 4,318) | <0.001 | 2013 |
| Rozenkiewicz | ESBL-producing K. pneumoniae** | Non-ESBL-producing K. pneumoniae | 61 | 112 | Hospitalisation cost   - Pharmacy - Antibiotic treatment - Nursery - Laboratory - Radiology - Inter consultations   Emergency room visits | Median (IQR): €6,718 (3,322 - 9,611) | Median (IQR): €3,688 (1,783 - 4,141), p < 0.001 | <0.001 | 2015 |
| *Estimated at national level in France: The number of visits to general practices for suspected UTIs was estimated to be 823,073 among over the age of 18 years in 2012 (95 % CI: 623,614–1,040,532). Among these clinical UTIs, 626,046 (95 % CI: 465,196–786,896) were confirmed by positive urine cultures, and 518,446 (95 % CI: 381,981–654,911) of these UTIs were due to E. coli. Among the E. coli-positive urine cultures, 38 % (95 % CI: 31–45 %) were resistant to at least one antibiotic (SDR, n = 197,009), and 19 % (15–24 %) were multi-resistant (MDR, n = 98,504). The number of wild type (susceptible) E. coli was estimated to be 222,932. | | | | | | | | | |
| **Abbreviations:** ESBL: Extended Spectrum Beta-Lactamase; IBL: Inducible Beta-Lactamases; GNB: Gram-negative bacteria; EK: Escherichia coli and Klebsiella species; E. coli: Escherichia coli; K. pneumoniae: Klebsiella pneumoniae; MDR: multi-drug resistant; SDR: single-drug resistant; OPAT: outpatient parenteral antimicrobial therapy | | | | | | | | | |

**Table S5 Quality assessment results**

The criteria for controlled before / after (CBA) study, controlled interrupted time-series (ITS) study, non-controlled ITS / BA study, and qualitative study are not presented as no such study design was identified in this review.

| **Study details** | **Study type** | **Methodology** | | | | | | | | | | | | | | | | | | | | **Overall** | | | |
| --- | --- | --- | --- | --- | --- | --- | --- | --- | --- | --- | --- | --- | --- | --- | --- | --- | --- | --- | --- | --- | --- | --- | --- | --- | --- |
|  |  | **All** | **RCT & CRCT** | | | | | **Cohort Studies** | | | | **All studies (quantitative and qualitative)** | | | | | | | | | |  |  |  |  |
|  |  | 1 | 2 | 3 | 4 | 5 | 6 | 18 | 18 | 19 | 20 | 25 | 26 | 27 | 28 | 29 | 30 | 31 | 32 | 33 | 34 | Final score | Minimum criteria met? | Minimum score met? | Related to aims of review? |
| Madrazo | Cohort | 2 |  |  |  |  |  | 2 | 2 | 2 | 2 | 2 |  | 2 | 2 | 2 | 2 | 2 | 2 | 2 | 2 | 28 | Yes | Yes | Yes |
| Mark | Cohort | 2 |  |  |  |  |  | 2 | 2 | 2 | 2 | 2 |  | 2 | 2 | 2 | 2 | 2 | 2 | 2 | 2 | 28 | Yes | Yes | Yes |
| Sozen | Cohort | 2 |  |  |  |  |  | 2 | 2 | 2 | 2 | 2 |  | 2 | 2 | 1 | 0 | 0 | 0 | 2 | 1 | 20 | Yes | Yes | Yes |
| Tabak | Cohort/case control | 2 |  |  |  |  |  | 2 | 2 | 2 | 2 | 2 |  | 2 | 2 | 2 | 2 | 2 | 2 | 1 | 1 | 26 | Yes | Yes | Yes |
| Wozniak | Cohort/case control | 2 |  |  |  |  |  | 2 | 2 | 2 | 2 | 2 |  | 2 | 2 | 1 | 2 | 2 | 2 | 2 | 2 | 27 | Yes | Yes | Yes |
| Chang | Cohort/case control | 2 |  |  |  |  |  | 2 | 2 | 2 | 2 | 2 |  | 2 | 2 | 1 | 2 | 0 | 2 | 1 | 1 | 23 | No | Yes | Yes |
| Cheong | Cohort/case control | 2 |  |  |  |  |  | 2 | 2 | 2 | 2 | 2 |  | 2 | 2 | 2 | 2 | 0 | 2 | 0 | 2 | 24 | Yes | Yes | Yes |
| François | Cohort | 2 |  |  |  |  |  | 1 | 1 | 2 | 2 | 2 |  | 2 | 2 | 0 | 2 | 2 | 2 | 1 | 2 | 23 | No | Yes | Yes |
| Kim | Cohort/case control | 2 |  |  |  |  |  | 2 | 2 | 2 | 2 | 1 |  | 2 | 2 | 2 | 2 | 0 | 2 | 1 | 1 | 23 | Yes | Yes | Yes |
| Little | RCT | 2 | 2 | 2 | 1 | 2 | 2 |  |  |  |  | 0 | 2 | 2 | 2 | 2 | 2 | 2 | 2 | 1 | 1 | 27 | Yes | Yes | Yes |
| Cardwell | Cohort | 2 |  |  |  |  |  | 2 | 2 | 2 | 2 | 2 |  | 2 | 2 | 2 | 2 | 2 | 2 | 2 | 1 | 27 | Yes | Yes | Yes |
| Esteve-Palau | Cohort/case control | 2 |  |  |  |  |  | 2 | 2 | 2 | 2 | 2 |  | 2 | 2 | 1 | 2 | 2 | 2 | 2 | 2 | 27 | No | Yes | Yes |
| MacVane | Cohort/case control | 2 |  |  |  |  |  | 2 | 2 | 2 | 2 | 2 |  | 2 | 2 | 1 | 2 | 2 | 2 | 2 | 2 | 27 | No | Yes | Yes |
| Rozenkiewicz | Cohort | 2 |  |  |  |  |  | 2 | 2 | 2 | 2 | 2 |  | 2 | 2 | 1 | 2 | 1 | 2 | 2 | 2 | 26 | No | Yes | Yes |
| Zilberberg | Cohort | 2 |  |  |  |  |  | 2 | 2 | 2 | 2 | 2 |  | 2 | 2 | 1 | 2 | 2 | 2 | 2 | 2 | 27 | No | Yes | Yes |
|  | | (Criteria ID) Scoring system: Yes = 2 points; Unclear = 1 point; No = 0 points | | | | | | | | | | | | | | | | | | | | | | | |

**Criteria**

1. Clearly stated aims?
2. Sequence generation
3. Allocation concealment
4. Blinding
5. Follow-up of professionals
6. Follow-up of patients or episodes of care
7. Comparability of groups (2E)

Comparability of outcomes (3G)

1. Sufficient follow-up period
2. Protection against information bias (5B)
3. Appropriate qualitative methodology?
4. Appropriate Study Design?
5. Sampling and Recruitment appropriate?
6. Data collection appropriate?
7. Blinded assessment of primary outcome measures? (protection against detection bias) (3E)
8. Intervention unlikely to affect data collection?
9. Reliable primary outcome measures? (3F)
10. Free of selective outcome reporting(7A)
11. Incomplete outcome data addressed? (4C)
12. Analysis sufficiently rigorous/ free of bias?
13. Limitations addressed?
14. Conclusions clear & justified?
15. Free of other risk of bias? Threats to internal/external validity? Researcher bias/reflexivity?
16. Ethical issues addressed?

**Figure S1: Funnel plot for publication bias in mortality**

Eggar’s test: p-value = 0.9699

**
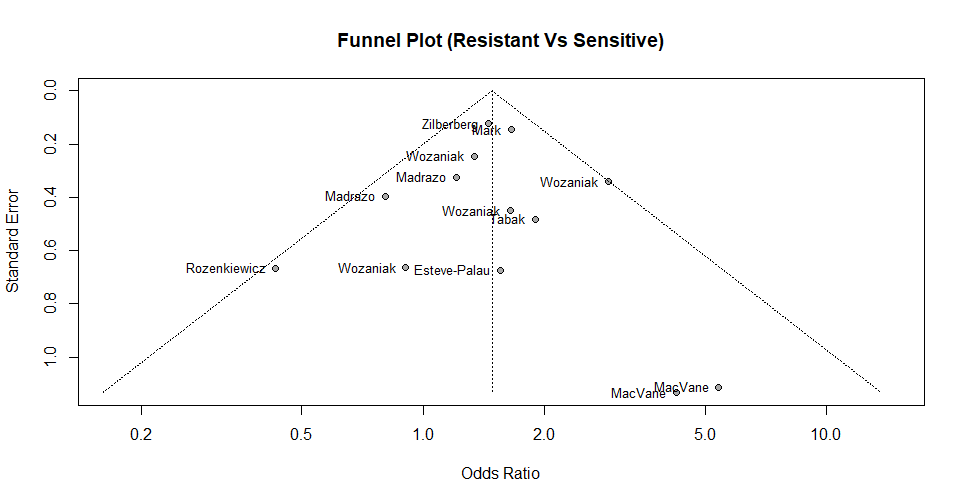
**
